# Trends in Treatment Choice for Early Glottic Cancer

**DOI:** 10.3390/jcm14093095

**Published:** 2025-04-30

**Authors:** Emily Y. Huang, Henry H. Joo, Sarek A. Shen, Matthew R. Naunheim, Ved Tanavde, Lee M. Akst

**Affiliations:** 1 Department of Otolaryngology—Head and Neck Surgery, Johns Hopkins University School of Medicine, Baltimore, MD 21287, USA; yhuan211@jhmi.edu (E.Y.H.); sshen21@jhmi.edu (S.A.S.); vedtanavde@jhmi.edu (V.T.); 2 Mass Eye and Ear, Department of Otolaryngology—Head and Neck Surgery, Boston, MA 02114, USA; mnaunheim@mgb.org

**Keywords:** early glottic cancer, cancer treatment choice, NCDB

## Abstract

**Background/Objective:** Systemic investigation into treatment for early-stage glottic cancer (EGC) has demonstrated similar functional and oncologic outcomes between transoral laser microsurgery (TLM) and external beam radiotherapy (XRT). In this study, we aim to identify longitudinal trends in treatment decisions and patient demographics. **Methods:** This is a retrospective longitudinal study on all cases of T1-2N0M0 glottic carcinoma queried from the NCDB between 2004 and 2017. The ratio of TLM to XRT (TLM/XRT) performed per year was calculated and further stratified by insurance status, education, income, and treatment facility location. Univariable and multivariable linear regressions were used to assess the trend of TLM/XRT over time and evaluate the effect of demographic characteristics on the TLM/XRT ratio. **Results:** A total of 38,428 EGC patients were analyzed: 2169 (5.6%) received TLM; 36,259 (94.4%) underwent XRT. The overall ratio of TLM/XRT increased over time from 0.04 in 2004 to 0.08 in 2017. Significant increases were observed in the higher-income quartiles (Q4: *p* < 0.001, Q3: *p* = 0.02, Q2 < 0.001) and among patients with private (*p* < 0.02) or public (*p* = 0.003) insurance. TLM/XRT rose significantly over time in the highest (Q4), third (Q3), and lowest (Q1) education quartiles but not in the second (Q2). Regionally, increases were observed in the Northeast (*p* < 0.001) and West (*p* = 0.008), with no significant change in the South or Midwest. By T stage, only T1 tumors showed a significant increase in TLM/XRT over time (*p* < 0.001). **Conclusions:** While the majority of patients receive XRT as the initial treatment for EGC, the proportion of TLM has been slowly increasing over time. Patient insurance status, education, income, facility geography, and T stage are correlated with increasing use of TLM.

## 1. Introduction

Early-stage glottic cancer (EGC) is a highly treatable disease that can be managed using various treatment modalities [1,2,3]. Transoral laser microsurgery (TLM) and external beam radiotherapy (XRT) are often recommended as the two primary therapeutic options, each as a single-modality treatment [2]. However, there remains debate on whether TLM or XRT is the superior treatment modality in terms of functional and oncologic outcomes. Some studies have demonstrated similar outcomes between the two treatment modalities [4,5]. However, there are systemic reviews that have suggested TLM to offer better overall survival, disease-specific survival, and laryngeal preservation in comparison to XRT [6,7,8]. TLM is also reported to have shorter hospital stays, faster recovery times, lower costs, and the ability to avoid the need for a permanent tracheostomy [9,10,11]. In a study investigating treatment preferences in patients with EGC, 96% of patients preferred TLM in comparison to radiotherapy [12]. Nonetheless, despite these reported advantages, TLM does have practical limitations that may affect its broader adoption. TLM requires specialized equipment and trained surgical professionals, which may not be universally available [12]. In certain cases, such as those with anterior commissure involvement, poor glottic exposure may render TLM infeasible, necessitating the use of radiotherapy [13].

Given the ongoing debate, opinions on the selection of optimal treatment strategies for early-stage glottic cancer in the past decades have continued to evolve with variability between specialties [14]. Hoffman et al. reported on the trends of laryngeal cancer demographics and treatment choices from 1985 to 2001; however, there have been no updated studies on the longitudinal evolution of EGC management modality [15].

Moreover, factors such as patient preference, clinical characteristics, and availability of both modalities at a particular clinical practice can also contribute to the complexity of treatment choice [6]. Some studies have identified socioeconomic factors associated with treatment choice including geography, income level, and insurance status [16]. Nonetheless, these cross-sectional studies are limited in analyzing the temporal relationship between patient sociodemographic factors and EGC primary treatment choices. To address this gap in the literature, the current study aims to identify trends in EGC primary treatment choices and patient demographics from 2004 to 2017 using the National Cancer Database (NCDB).

## 2. Materials and Methods

### 2.1. Survey and Participants

The National Cancer Database (NCDB) is headed by the American College of Surgeons Commission on Cancer and the American Cancer Society. The NCDB accumulates data from over 1500 cancer programs, encompassing more than 70% of newly diagnosed cancer cases in the United States [17]. The cohort for this retrospective longitudinal analysis includes all patients who received either primary XRT or TLM treatment for T1-2N0M0 glottic carcinoma between 2004 and 2017. Patients with carcinoma in situ (Tis), severe dysplasia, and recurrent disease were excluded. Additionally, patients who received both XRT and TLM were excluded due to inability to determine the initial primary treatment modality for analysis. Approval for this study was obtained from the Institutional Review Board at Johns Hopkins University.

The primary outcome in this study was the yearly ratio of EGC cases that were managed with surgery to those that were managed with radiotherapy. This ratio (TLM/XRT) was simply calculated by dividing the number of surgery cases by the number of radiotherapy cases for each year between 2004 and 2017. For further analysis, we calculated this ratio for subsets of the original database stratified by certain demographic characteristics including income, insurance, education, and facility location. Income and education were estimated by matching the patient’s zip code recorded at the time of diagnosis with the U.S. Census and American Community Survey, respectively [18]. The education level was assigned using a zip code-level proxy based on the percentage of adult residents with a high school diploma. Insurance types were divided into public, private, and uninsured groups, and facility locations were aggregated into Northeast, Midwest, South, and West based on the U.S. Census region definitions [19].

### 2.2. Statistical Analysis

The demographic characteristics of the patients receiving TLM versus XRT were compared using chi-square tests except for age, which was evaluated using a Wilcoxon rank sum test. A simple linear regression was used to assess the overall trend of the surgery-to-radiotherapy ratio over time. To examine subgroup-specific temporal trends, we stratified by each variable of interest and conducted multiple univariable linear regressions to assess the association between TLM/XRT ratio and year. All the analyses were conducted in R (version 4.2.1), and significance was determined using a Bonferroni-adjusted alpha level of *p* = 0.006.

## 3. Results

A total of 38,428 adult patients with early-stage glottic cancer who underwent either primary XRT (n = 36,259; 94.4%) or TLM (n = 2169; 5.6%) were identified from the NCDB database for the years 2004 to 2017. Overall, 81% of the patients were male; 86% identified as white; 61% had public insurance; 79% resided in a metropolitan area; 20% were in the lowest income quartile; and 18% were in the lowest high school diploma quartile. The average patient age was 66 years old. Additional patient characteristics of the two primary treatment groups are presented in Table 1.

**Figure 1:**
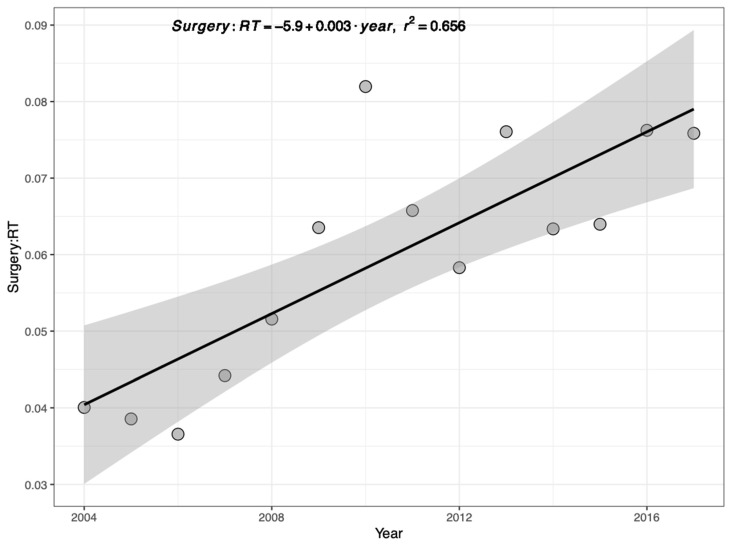
Linear regression plot of TLM/XRT performed by year. The shaded region represents the 95% confidence interval.

**Figure 2:**
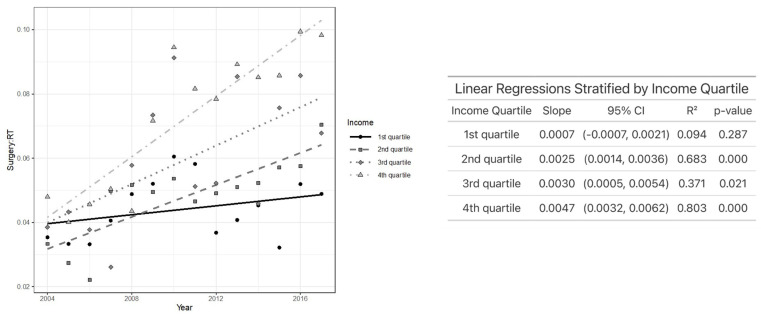
Plot of TLM/XRT performed each year by income (**left**) and linear regression analysis showing the association between TLM/XRT ratio and year after stratifying by income (**right**).

Figure 1 represents the ratio of primary TLM and XRT performed per year, which has gradually increased over time from 0.04 in 2004 to 0.076 in 2017. Further analysis of TLM/XRT over time was subdivided by income, insurance, education, and facility location. Among the income quartiles, the TLM/XRT ratio increased most rapidly in the highest (fourth) quartile and least in the lowest (first) quartile. Specifically, patients in the second (*p* < 0.001), third (*p* = 0.02), and fourth (*p* < 0.001) income quartiles experienced a statistically significant increase in TLM/XRT over time, while those in the first quartile did not (Figure 2).

**Figure 3:**
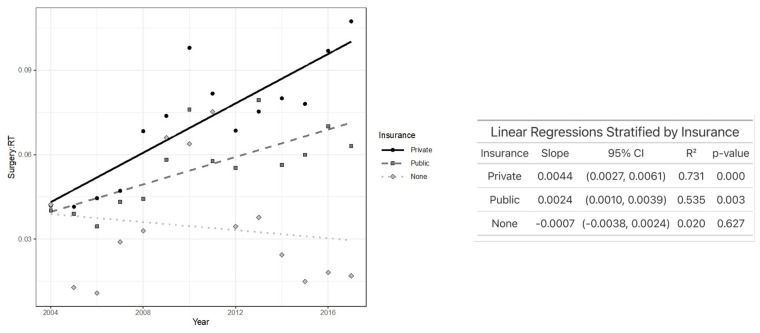
Plot of TLM/XRT performed each year by insurance status (**left**) and linear regression analysis showing the association between TLM/XRT ratio and year after stratifying by insurance status (**right**).

Figure 3 depicts that TLM/XRT increased the most over time in patients with private insurance, and it moderately increased over time in those with public insurance. However, TLM/XRT decreased over time in those with no insurance, indicating that increasingly fewer TLMs are being performed in comparison to XRT throughout the years. Patients with private insurance (*p* < 0.02) and public insurance (*p* = 0.003) experienced a statistically significant increase in TLM/XRT over time, while no significant change over time was observed among uninsured patients.

**Figure 4:**
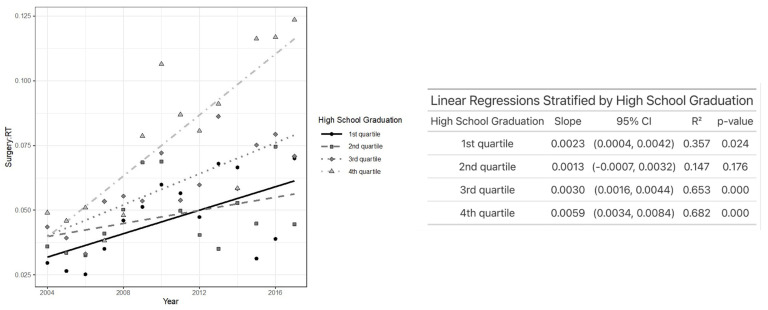
Plot of TLM/XRT performed each year by education status (**left**) and linear regression analysis showing the association between TLM/XRT ratio and year after stratifying by education status (**right**).

**Figure 5:**
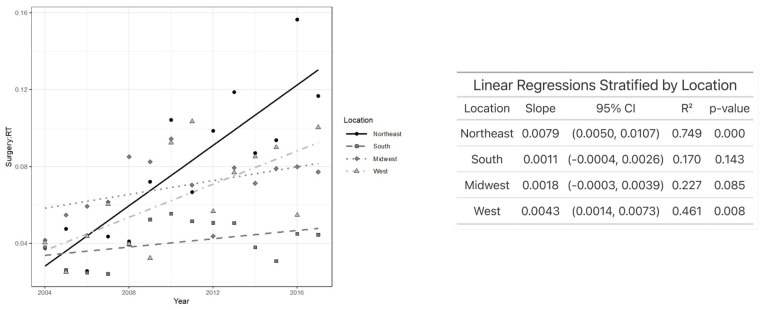
Plot of TLM/XRT performed each year by facility location (**left**) and linear regression analysis showing the association between TLM/XRT ratio and year after stratifying by facility location (**right**).

**Figure 6:**
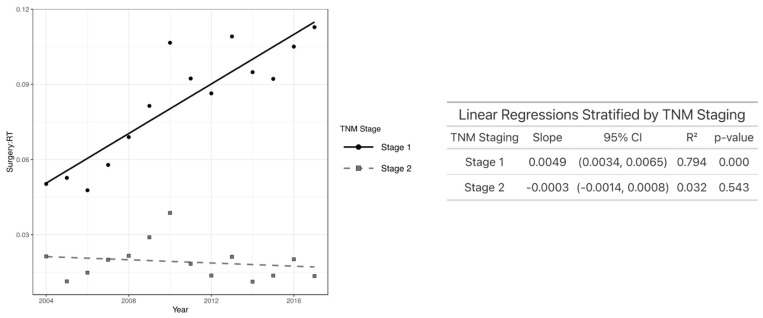
Plot of TLM/XRT performed each year by T stage (**left**) and linear regression analysis showing the association between TLM/XRT ratio and year after stratifying by T stage (**right**).

Regarding education, patients in the highest education quartiles experienced the greatest increase in TLM/XRT over time. Specifically, patients in the third (*p* < 0.001), fourth (*p* < 0.001), and even the lowest (first) quartile (*p* < 0.02) showed a significant increase in TLM/XRT, while those in the second quartile did not demonstrate a significant change over time (Figure 4). As demonstrated in Figure 5, individuals who received primary treatment from a facility in the Northeast saw the most rapid increase in TLM/XRT over time. A statistically significant increase was observed in the Northeast (*p* < 0.001) and the West (*p* = 0.008), while no significant change was noted among patients treated in the South or Midwest. In Figure 6, patients with T1 glottic cancer (*p* < 0.001) exhibited a significant increase in TLM/XRT over time, while those with T2 tumors did not experience a significant change.

## 4. Discussion

In this analysis of trends in EGC treatment choice and demographic characteristics from 2004 to 2017 using the NCDB, we observed a gradual increase in TLM/XRT performed as the primary treatment modality. Over that period, patients with higher income, higher education, private insurance, and treatment received from facilities located in the Northeast saw the most rapid increase in TLM/XRT. While most subgroups (income level, education, insurance status, facility location) saw an increase in TLM/XRT over time, patients with no insurance experienced a decrease in TLM/XRT.

Although both XRT and TLM are acceptable treatment options for early glottic cancer, systematic reviews have reported TLM to have better overall survival, disease-specific survival, and laryngeal preservation in comparison to XRT [6,7,8]. The increase in TLM/XRT in the past two decades indicates a positive trend toward performing more TLM in comparison to XRT. This is likely due to lower treatment costs, shorter hospital stays, and faster recovery times [11,20,21]. Additionally, the observed rise in TLM may also reflect broader workforce trends, including an increase in the number of fellowship-trained head and neck surgeons and laryngologists over the study period, which may have expanded access to TLM and influenced treatment patterns [22,23]. At the same time, advances in radiotherapy techniques, such as single vocal cord irradiation (SVCI), have emerged as promising alternatives in select U.S. centers. SVCI employs a mild hypofractionated regimen with conformal targeting to reduce radiation exposure to surrounding structures, while maintaining effective oncologic control [24]. Although not yet widespread, this technique may offer future advantages and warrants continued evaluation.

Yet, despite these advancements, our findings suggest that the increase in TLM utilization has not been equitably distributed across all patient groups. The present analyses demonstrate that the increase in TLMs performed is primarily driven by particular subgroups of patients with higher socioeconomic status, while those patients with lower income, less education, no insurance, or public insurance experience a relatively limited increase or even decrease in TLMs performed compared with XRT from 2004 to 2017.

This widening gap in the growth of TLM/XRT between certain patient demographics is important to highlight. Within our study, patients with higher socioeconomic status were more likely to opt for surgical treatment of their early glottic cancer. Plausible explanations for this trend include disparities in access to specialist care and barriers to early cancer diagnosis. Specialist providers are more likely to be localized to city centers where wait time and distance can be potential barriers for patients with lower socioeconomic status [25,26]. Furthermore, publicly insured and uninsured patients are also at increased risk for presenting with advanced laryngeal cancer due to lesser healthcare access and utilization [27]. One study reported that lower socioeconomic status was associated with more-advanced glottic cancer presentation, lower cause-specific survival, and higher locoregional failure. Surprisingly, we found that patients with no insurance were more likely to undergo primary XRT in comparison to TLM despite the lower estimated total costs of TLM [20,28,29]. A survey of the out-of-pocket cost (OOPC) for head and neck cancer treatments found that the OOPC of surgery alone was significantly lower than the OOPC of chemoradiation and radiotherapy [30]. Additional studies are needed to further investigate additional barriers to TLM for uninsured individuals or those with public insurance.

In previous investigations of the temporal trends in EGC treatment choices, Friedman et al. and Mourad et al. did not find an association between education and treatment decision [16,31]. Conversely, we found that different education levels had significantly different rates in TLM/XRT, after controlling for the year of diagnosis. Furthermore, we demonstrated that patients in the highest quartile of high school graduation saw the greatest increase in TLM/XRT from 2004 to 2017. The differences in the findings could be plausibly attributed to differences in education status among the surgery candidates or a small TLM sample size.

Our findings revealed regional differences in EGC treatment choice, with treatment facilities in the Northeast and West experiencing a significant increase in TLM/XRT over time. Other cross-sectional studies have reported the increased use of surgery for EGC in the West with the NCDB database and in the South using the SEER database [16,31]. These differences in findings indicate that potential regional differences exist in surgical approach (TLM and open partial laryngectomy) for EGC. The increased use of TLM in the Northeast could be attributed to regional biases in training programs or the higher density of academic teaching centers [32].

Additionally, when stratifying by T stage, we found that patients with T1 glottic cancer had a significant increase in TLM/XRT per year, whereas those with T2 tumors did not experience a significant increase in TLM/XRT per year. This finding aligns with the international trends for the treatment of T1 glottic cancer. For example, in the Netherlands, approximately 58% of patients with Tcis–T1b glottic tumors are treated with TLM, which reflects a similarly increased preference for the surgical management of T1-stage disease [33]. This parallel suggests that the observed rise in TLM/XRT for T1 glottic cancers in our cohort may reflect broader global patterns in clinical practice favoring TLM for early-stage lesions [34]. In addition, the greater increase in TLM utilization among T1 tumors may also reflect improvements in early detection and diagnosis over time, enabling more patients to qualify for surgical management [22].

These findings raise broader clinical and policy implications, particularly regarding equitable access to guideline-concordant care. As socioeconomic disparities appear to influence access to surgical treatment, this warrants careful consideration in healthcare planning and policy development.

To our knowledge, this is the largest study to date of longitudinal trends in early-stage glottic cancer treatment selection and associated patient demographics. However, a few limitations exist. First, some patients were excluded from analysis due to missing staging and demographic data within the NCDB. Second, the temporal nature of the analyses resulted in relatively small sample sizes per year, prohibiting the inclusion of race/ethnicity and residency type (e.g., rural, metropolitan) as sub-analysis variables due to small intersections between demographics and treatment groups. One cross-sectional study reported no significant association between race/ethnicity with EGC treatment selection and a significantly greater likelihood of pursuing surgical treatment when living in a metropolitan area [16]. Future studies could consider additional approaches to gain a deeper understanding of the association of these variables with TLM/XRT trends longitudinally. Third, additional factors that could affect treatment choices including clinical characteristics, availability of either TLM or XRT at facility, and distance to the nearest treatment facility could not be accounted for due to inherent limitations of the database. Most notably, the patients were more likely to believe that the practitioner they saw first (surgeons, radiation oncologist, or medical oncologist) was the most important factor in treatment decision-making for laryngeal cancer [35]. Future studies incorporating institutional treatment protocols and multidisciplinary decision-making data may help further contextualize the observed variation in treatment selection. Finally, it is likely that the patient demographic variables included in this study such as income level, insurance status, and education are inextricably associated with each other. This could potentially complicate the interpretation of individual associations in longitudinal treatment choice trends. To preserve interpretability and clearly visualize subgroup-specific trends over time, we performed stratified univariable regressions. While multivariable modeling could adjust for confounding, it would have reduced sensitivity to year-by-year variation and shifted the analytical focus from population-level trends to individual-level associations—an important direction for future research, but beyond the scope of this study.

## 5. Conclusions

This study examined the evolving trends in patient demographics and treatment decisions for early-stage glottic cancer using TLM and XRT. Our findings demonstrate the gradual increase in TLM compared to XRT performed in the past two decades. Certain sociodemographic disparities such as income level, insurance status, education status, and facility location may have played important roles in EGC treatment selection. These findings will shed light on the factors influencing treatment decisions, potential disparities in care, and the impact of patient demographics on treatment choices.

## Figures and Tables

**Table 1:** Patient characteristics.

Characteristic	Overall, n= 38,428 ^1^	XRT, n = 36,259 ^1^	TLM, n = 2169 ^1^	*p*-Value ^2^
**Sex**				0.018
Male	31,150 (81%)	29,350 (81%)	1800 (83%)	
Female	7278 (19%)	6909 (19%)	369 (17%)	
**Age [Mean (SD)]**	66 (11)	66 (11)	66 (12)	0.041
**Race**				<0.001
White	32,980 (86%)	31,096 (86%)	1884 (87%)	
Black	4335 (11%)	4147 (11%)	188 (8.7%)	
Other	1113 (2.9%)	1016 (2.8%)	97 (4.5%)	
**Ethnicity**				0.2
Non-Hispanic	34,880 (91%)	32,928 (91%)	1952 (90%)	
Hispanic	3548 (9.2%)	3331 (9.2%)	217 (10%)	
**Insurance status**				<0.001
Public	23,535 (61%)	22,288 (61%)	1247 (57%)	
Private	13,043 (34%)	12,183 (34%)	860 (40%)	
None	1222 (3.2%)	1179 (3.3%)	43 (2.0%)	
Unknown	628 (1.6%)	609 (1.6%)	19 (0.9%)	
**Income**				<0.001
1st quartile	7628 (20%)	7307 (20%)	321 (15%)	
2nd quartile	8463 (22%)	8079 (22%)	384 (18%)	
3rd quartile	8082 (21%)	7632 (21%)	450 (21%)	
4th quartile	10,042 (26%)	9369 (26%)	673 (31%)	
Unknown	4213 (11%)	3872 (11%)	341 (16%)	
**Residency**				0.002
Metro	30,427 (79%)	28,719 (81%)	1708 (79%)	
Urban	6119 (16%)	5835 (16%)	284 (13%)	
Rural	854 (2.2%)	820 (2.3%)	34 (1.6%)	
Unknown	1028 (2.7%)	885 (2.4%)	143 (6.6%)	
**High School Diploma Rate**				<0.001
1st quartile	7876 (20%)	7526 (21%)	350 (16%)	
2nd quartile	9961 (26%)	9507 (26%)	454 (21%)	
3rd quartile	9380 (24%)	8857 (24%)	523 (24%)	
4th quartile	7083 (18%)	6577 (18%)	506 (23%)	
Unknown	4128 (11%)	3792 (10%)	336 (15%)	
**Facility Location**				<0.001
Northeast	8006 (21%)	7420 (20%)	586 (27%)	
South	14,930 (39%)	14,343 (40%)	587 (27%)	
Midwest	10,632 (28%)	9939 (27%)	693 (32%)	
West	4502 (12%)	4233 (12%)	269 (12%)	
Unknown	358 (0.9%)	324 (0.9%)	34 (1.6%)	

^1^ n (%); Mean (SD); ^2^ Pearson’s chi-squared test; Wilcoxon rank sum test; RT = radiotherapy.

## Data Availability

The data used in this study were obtained from the National Cancer Database (NCDB) and are not publicly available. Access is restricted to approved institutions through the American College of Surgeons.

## Abbreviations

The following abbreviations are used in this manuscript:

EGC	Early glottic cancer
TLM	Transoral laser microsurgery
XRT	External beam radiotherapy
NCDB	National Cancer Database
OOPC	Out-of-pocket cost

## References

[1] Hartl D.M., Brasnu D.F. (2015). Contemporary Surgical Management of Early Glottic Cancer. Otolaryngol. Clin. N. Am..

[2] Hartl D.M., Ferlito A., Brasnu D.F., Langendijk J.A., Rinaldo A., Silver C.E., Wolf G.T. (2011). Evidence-Based Review of Treatment Options for Patients with Glottic Cancer. Head Neck.

[3] Allegra E., Saita V., Azzolina A., De Natale M., Bianco M.R., Modica D.M., Garozzo A. (2018). Impact of the Anterior Commissure Involvement on the Survival of Early Glottic Cancer Treated with Cricohyoidoepiglottopexy: A Retrospective Study. Cancer Manag. Res..

[4] Abdurehim Y., Hua Z., Yasin Y., Xukurhan A., Imam I., Yuqin F. (2012). Transoral Laser Surgery versus Radiotherapy: Systematic Review and Meta-Analysis for Treatment Options of T1a Glottic Cancer. Head Neck.

[5] Warner L., Lee K., Homer J.J. (2017). Transoral Laser Microsurgery versus Radiotherapy for T2 Glottic Squamous Cell Carcinoma: A Systematic Review of Local Control Outcomes. Clin. Otolaryngol..

[6] Vaculik M.F., MacKay C.A., Taylor S.M., Trites J.R.B., Hart R.D., Rigby M.H. (2019). Systematic Review and Meta-Analysis of T1 Glottic Cancer Outcomes Comparing CO_2_ Transoral Laser Microsurgery and Radiotherapy. J. Otolaryngol. Head Neck Surg..

[7] Guimarães A.V., Dedivitis R.A., Matos L.L., Aires F.T., Cernea C.R. (2018). Comparison between Transoral Laser Surgery and Radiotherapy in the Treatment of Early Glottic Cancer: A Systematic Review and Meta-Analysis. Sci. Rep..

[8] Mo H.L., Li J., Yang X., Zhang F., Xiong J.W., Yang Z.L., Tan J., Li B. (2017). Transoral Laser Microsurgery versus Radiotherapy for T1 Glottic Carcinoma: A Systematic Review and Meta-Analysis. Lasers Med. Sci..

[9] Goor K.M., Peeters A.J.G.E., Mahieu H.F., Langendijk J.A., Leemans C.R., Verdonck-De Leeuw I.M., Van Agthoven M. (2007). Cordectomy by CO_2_ Laser or Radiotherapy for Small T1a Glottic Carcinomas: Costs, Local Control, Survival, Quality of Life, and Voice Quality. Head Neck.

[10] Nasef H.O., Thabet H., Piazza C., Del Bon F., Eid M., Banna M.E., Nicolai P. (2016). Prospective Analysis of Functional Swallowing Outcome after Resection of T2 Glottic Carcinoma Using Transoral Laser Surgery and External Vertical Hemilaryngectomy. Eur. Arch. Oto-Rhino-Laryngol..

[11] Sjögren E. (2017). Transoral Laser Microsurgery in Early Glottic Lesions. Curr. Otorhinolaryngol. Rep..

[12] Hans S., Baudouin R., Circiu M.P., Couineau F., Lisan Q., Crevier-Buchman L., Lechien J.R. (2022). Laryngeal Cancer Surgery: History and Current Indications of Transoral Laser Microsurgery and Transoral Robotic Surgery. J. Clin. Med..

[13] Yin Y., Cai Q., Zheng Y., Huang X., Peng J., Liang F., Yang J., Chen W., Su Y., Guan Z. (2023). CO_2_ Transoral Laser Microsurgery for Early Glottic Carcinoma with Anterior Commissure Involvement. Auris Nasus Larynx.

[14] Makki F.M., Williams B., Rajaraman M., Hart R.D., Trites J., Brown T., Taylor S.M. (2016). Current Practice Patterns in the Management of Glottic Cancer in Canada: Results of a National Survey. J. Otolaryngol. Head Neck Surg..

[15] Hoffman H.T., Porter K., Karnell L.H., Cooper J.S., Weber R.S., Langer C.J., Ang K.-K., Gay G., Stewart A., Robinson R.A. (2006). Laryngeal Cancer in the United States: Changes in Demographics, Patterns of Care, and Survival. Laryngoscope.

[16] Friedman A.D., Gengler I., Altaye M., Tabangin M.E. (2022). Early-Stage Glottic Carcinoma in the United States: Demographics and Treatment Choice. Laryngoscope.

[17] About the National Cancer Database|ACS. https://www.facs.org/quality-programs/cancer-programs/national-cancer-database/about/.

[18] National Cancer Database Participant User File. https://www.facs.org/media/brilfbgu/puf-2020-data-dictionary.pdf.

[19] United States Census Bureau Geographic Levels. https://www.census.gov/programs-surveys/economic-census/guidance-geographies/levels.html#par_textimage_34.

[20] Phillips T.J., Sader C., Brown T., Bullock M., Wilke D., Trites J.R.B., Hart R., Murphy M., Taylor S.M. (2009). Transoral Laser Microsurgery versus Radiation Therapy for Early Glottic Cancer in Canada: Cost Analysis. J. Otolaryngol. Head Neck Surg..

[21] Grant D.G., Salassa J.R., Hinni M.L., Pearson B.W., Hayden R.E., Perry W.C. (2007). Transoral Laser Microsurgery for Untreated Glottic Carcinoma. Otolaryngol. Head Neck Surg..

[22] Panuganti B.A., Stuart E., Weissbrod P. (2021). Changes in Treatment Trends in the Early Glottic Cancer Population after the Affordable Care Act. Head Neck.

[23] Talwar A., Gordon A.J., Bewley A.F., Fancy T., Lydiatt W.M., Weed D., Moore M.G., Givi B. (2022). Distribution of the Head and Neck Surgical Oncology Workforce in the United States. Head Neck.

[24] Levendag P.C., Teguh D.N., Keskin-Cambay F., Al-Mamgani A., van Rooij P., Astreinidou E., Kwa S.L.S., Heijmen B., Monserez D.A., Osman S.O.S. (2011). Single Vocal Cord Irradiation: A Competitive Treatment Strategy in Early Glottic Cancer. Radiother. Oncol..

[25] Lueckmann S.L., Hoebel J., Roick J., Markert J., Spallek J., von dem Knesebeck O., Richter M. (2021). Socioeconomic Inequalities in Primary-Care and Specialist Physician Visits: A Systematic Review. Int. J. Equity Health.

[26] Khalil D., Corsten M.J., Holland M., Balram A., McDonald J.T., Johnson-Obaseki S. (2018). Does Socioeconomic Status Affect Stage at Presentation for Larynx Cancer in Canada’s Universal Health Care System?. Otolaryngol. Head Neck Surg..

[27] Chen A.Y., Schrag N.M., Halpern M., Stewart A., Ward E.M. (2007). Health Insurance and Stage at Diagnosis of Laryngeal Cancer: Does Insurance Type Predict Stage at Diagnosis?. Arch. Otolaryngol. Head Neck Surg..

[28] Prettyjohns M., Winter S., Kerawala C., Paleri V., Robinson M., Bhide S., Capel M., Cox L., Fenlon M., Newman L. (2017). Transoral Laser Microsurgery versus Radiation Therapy in the Management of T1 and T2 Laryngeal Glottic Carcinoma: Which Modality Is Cost-Effective within the UK?. Clin. Otolaryngol..

[29] Halkud R., Chavan P., Shenoy A.M., Sharma V., Ranganath N., Pasha T., Shenoy P., Ravikumar B., Narayana S.M., Sharif M.I. (2017). Transoral Laser Microsurgery vs Radiotherapy for Early Glottic Cancer: Study at Tertiary Care Center in India. Int. J. Head Neck Surg..

[30] Khan M.N., Hueniken K., Manojlovic-Kolarski M., Eng L., Mirshams M., Khan K., Simpson C., Au M., Liu G., Xu W. (2022). Out-of-pocket Costs Associated with Head and Neck Cancer Treatment. Cancer Rep..

[31] Mourad M., Dezube A., Moshier E., Shin E. (2016). Geographic Trends in Management of Early-Stage Laryngeal Cancer. Laryngoscope.

[32] Smith D.H., Case H.F., Quereshy H.A., Mecham J.C., Bowe S.N., Carlson M.L., Cordero J. (2023). Geographic Distribution of Otolaryngology Training Programs and Potential Opportunities for Strategic Program Growth. Laryngoscope.

[33] Dorr M.C., Sewnaik A., Andrinopoulou E., Berzenji D., Dronkers E.A.C., Bernard S.E., Hoesseini A., Tans L., Rizopoulos D., Baatenburg de Jong R.J. (2023). Longitudinal Patient-Reported Voice Quality in Early-Stage Glottic Cancer. Otolaryngol. Head Neck Surg..

[34] Hendriksma M., van Loon Y., Klop W.M.C., Hakkesteegt M.M., Heijnen B.J., El Hasnaoui I., de Jong M., Langeveld T.P.M., van Benthem P.P.G., de Jong R.J. (2019). Quality of Life and Voice Outcome of Patients Treated with Transoral CO_2_ Laser Microsurgery for Early Glottic Carcinoma (T1–T2): A 2-Year Follow-up Study. Eur. Arch. Oto-Rhino-Laryngol..

[35] Shuman A.G., Larkin K., Thomas D., Palmer F.L., Fins J.J., Baxi S.S., Lee N., Shah J.P., Fagerlin A., Patel S.G. (2017). Patient Reflections on Decision Making for Laryngeal Cancer Treatment. Otolaryngol. Head Neck Surg..

